# Acute Management of Refractory Bullous Pemphigoid Using Plasma Exchange and Granulocyte-Monocyte-Apheresis: A Case Report

**DOI:** 10.1159/cde/abvag011

**Published:** 2026-08-24

**Authors:** Georgiana Ciobanu, Erich Reichinger, Ieva Saulite, Antonio Cozzio, Norbert Arn

**Affiliations:** a Health Ostschweiz, Cantonal Hospital of St. Gallen, HOCH, Clinic of Nephrology and Transplant Medicine, Apheresis Center, St. Gallen, Switzerland; b Department of Internal Medicine, Ente Ospedaliero Cantonale (EOC), Ospedale Regionale di Locarno “La Carità”, Locarno, Switzerland; c Health Ostschweiz, Cantonal Hospital of St. Gallen, HOCH, Department of Internal Medicine, St. Gallen, Switzerland; d Health Ostschweiz, Cantonal Hospital of St. Gallen, HOCH, Clinic of Dermatology, St. Gallen, Switzerland

**Keywords:** Bullous pemphigoid, Plasma exchange, Intravenous immunoglobulin, Autoimmune bullous diseases, Granulocyte and monocyte apheresis, Case report

## Abstract

**Introduction:**

Bullous pemphigoid (BP) is a chronic autoimmune blistering disorder, characterized by subepidermal blister formation and intense pruritus, predominantly affecting older adults. Its pathogenesis is complex and only partially understood, involving both humoral and cellular immune mechanisms. Current treatment strategies rely primarily on topical or systemic corticosteroids, depending on disease extent. However, the long-term use of corticosteroids is often constrained by substantial toxicity, prompting the use of steroid-sparing agents such as methotrexate, rituximab, and other immunomodulatory therapies. Nevertheless, the management of BP remains challenging, particularly in refractory cases, due to frequent relapses and treatment-associated adverse events.

**Case Presentation:**

We report the case of a 67-year-old woman with long-standing refractory BP and multiple relapses despite various therapeutic interventions, including dupilumab. Due to persistent disease activity, therapeutic plasma exchange was performed, resulting in marked clinical improvement and allowing subsequent maintenance therapy with intravenous immunoglobulin (IVIg). Six months later, however, she developed a severe flare with progressive blistering that remained refractory to high-dose systemic corticosteroids. Following repeat plasma exchange and subsequent granulocyte-monocyte apheresis (GMA), a marked clinical response was observed, with achievement of sustained clinical remission.

**Conclusion:**

In this case of severe refractory BP, sequential extracorporeal therapies were temporally associated with disease control. Plasma exchange followed by IVIg was associated with an initial clinical response, whereas repeat plasma exchange and subsequent GMA coincided with remission during a later inflammatory relapse. Although a causal relationship cannot be established from a single case, these observations suggest that tailored extracorporeal approaches including GMA may represent a valuable therapeutic option in selected patients with severe, treatment-refractory BP and warrant further investigation.

## Introduction

Bullous pemphigoid (BP) is a chronic autoimmune disorder characterized by subepidermal blister formation. Clinically, it presents with large, tense, fluid-filled blisters arising on normal or erythematous skin and is frequently accompanied by intense pruritus [1]. The disease predominantly affects individuals over 60 years of age [2, 3].

BP is mediated by autoantibodies directed against structural components of the dermal-epidermal junction, particularly BP180 and BP230, leading to complement activation and recruitment of inflammatory cells [4]. The chronic course of BP may result in recurrent skin damage, secondary infections, and a substantial reduction in quality of life [5].

The pathophysiology of BP is complex and remains incompletely understood. Both humoral and cellular immune mechanisms contribute to disease development, with increasing evidence supporting an important role for innate immune activation and autoinflammatory pathways. Following autoantibody binding and complement activation, mast cell degranulation and the recruitment of neutrophils, eosinophils, basophils, and macrophages promote tissue injury and subepidermal blister formation. In particular, activated neutrophils release proteolytic enzymes and inflammatory mediators that contribute directly to dermal-epidermal separation, providing a potential rationale for therapies targeting myeloid cell populations [2, 4, 5].

First-line treatment of localized BP consists of high-potency topical corticosteroids, whereas more extensive disease generally requires systemic corticosteroid therapy. However, the long-term use of corticosteroids is often limited by significant adverse effects, and corticosteroid-sparing immunosuppressive agents are frequently required. Refractory cases may necessitate advanced therapies, including biologic agents such as rituximab or intravenous immunoglobulin (IVIg). Despite these therapeutic options, the management of severe BP remains challenging, highlighting the need for safer and more effective long-term treatment strategies [6].

Adsorptive granulocyte-monocyte apheresis (GMA) is an extracorporeal therapy that selectively removes activated granulocytes and monocytes from the peripheral circulation using cellulose acetate bead columns. Activated neutrophils expressing Mac-1 (CD11b/CD18) are adsorbed to the beads through interactions involving complement fragment iC3b, thereby reducing the burden of circulating pro-inflammatory myeloid cells [7, 8]. Given the emerging evidence supporting a central role for neutrophils and other innate immune cells in BP pathogenesis, including their contribution to dermal-epidermal separation and blister formation, GMA may represent a biologically plausible adjunctive therapeutic strategy in refractory disease. Nevertheless, its role in BP has not been established and warrants further investigation.

We report a case of severe, refractory BP with multiple relapses highlighting the potential role of extracorporeal therapies, including GMA. This case report was prepared in accordance with the CARE (CAse REport) guidelines (see online suppl. material; for all online suppl. material, see https://doi.org/10.1159/cde/abvag011).

## Case Presentation

A 67-year-old woman was admitted to the intensive care unit in September 2023 following small bowel resection and ileostomy for an iatrogenic small bowel perforation that occurred during cholecystectomy and was complicated by septic shock. Her medical history was notable for urothelial carcinoma, rectal carcinoma, and BP, diagnosed in May 2023.

During her intensive care unit stay from October to November 2023, BP manifestations, particularly painful blistering, progressed markedly (Fig. 1a). Oral prednisone was initiated in November at 20 mg/day and reduced to 7.5 mg/day 4 days later. Following transfer to the geriatrics department, prednisone was gradually tapered and subsequently discontinued. The sequence of therapeutic interventions is summarized in Table 1.

**Fig. 1.**
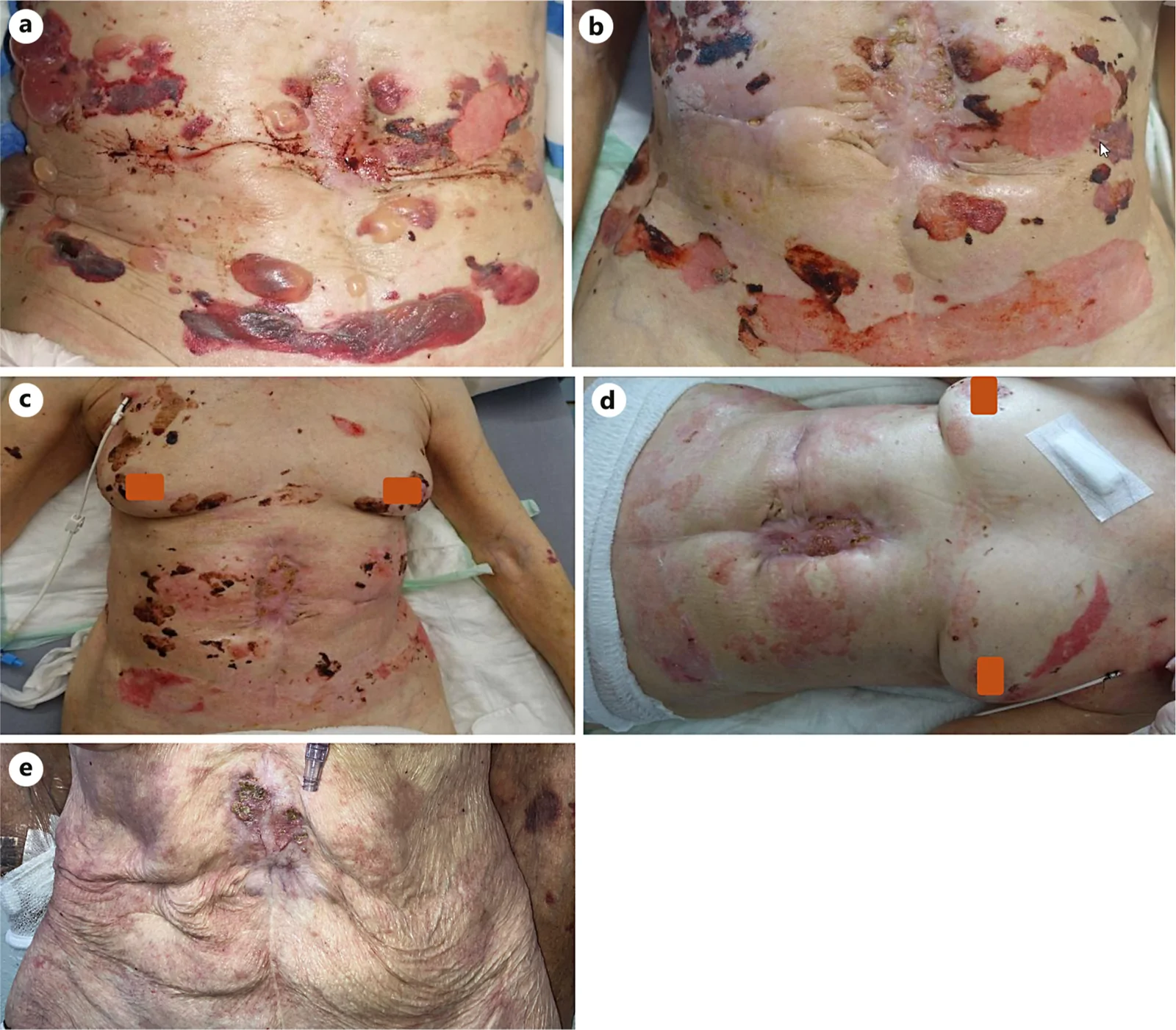
BP: clinical course and skin manifestations during treatment. Skin findings before initiation of therapeutic plasma exchange (TPE) (**a**); after two TPE sessions (**b**); 6 days after completion of the fifth (final) TPE session (**c**); and 4 weeks after the initial TPE course (**d**). skin findings after three TPE sessions during the relapse, followed by three granulocyte-monocyte apheresis (GMA) sessions (**e**). Archive: Photo Documentation, Cantonal Hospital St. Gallen HOCH, St. Gallen, Switzerland.

**Table 1.** Sequence of therapeutic interventions

Period	Clinical status	Therapeutic strategies
November 2023	First admission	Oral prednisone (20 mg/day)
December 2023	Second admission	Mometasone furoate and dimetindene
		Oral and IV antihistamines
		Topical steroids
		Oral prednisone (40 mg/day)
February 2024	First relapse	Start dupilumab 300 mg SC every 2 weeks
		Plasma exchange
		*Spectra Optia; 1–1.5 plasma volumes/session, total of 5 sessions*
End of February 2024	Good clinical response	IVIg (cycle 1, 20 g/day for 5 days
April 2024	Rapid remission	IVIg (cycle 2, 20 g/day for 5 days
September 2024	Severe flare	Oral prednisolone 50 mg/day for 5 days
	Second relapse	Plasma exchange
		*Spectra Optia 1–1.5 plasma volumes/session, 3 sessions*
End of September 2024	Suspected bacterial superinfection	Co-amoxicillin 2.2 g IV three times daily
October 2024	Severe blistering	GMA: 3 sessions
		*Adacolumn; 180 min/session; heparin 5000 IU at 6 mL/h*
November 2024 onward	Sustained remission	Dupilumab 300 mg SC every 2 weeks

IV, intravenous; IVIg, intravenous immunoglobulin; SC, subcutaneous; g, grams; mg, milligrams; GMA, granulocyte-monocyte apheresis.

The patient was later transferred to a rehabilitation clinic. Her clinical course was complicated by recurrent episodes of peristomal dermatitis requiring rehospitalization. Despite treatment with topical mometasone furoate, dimetindene, and oral and intravenous antihistamines, her condition worsened, with progressive erythematous plaques, blistering, pain, and pruritus. Systemic therapy was intensified with prednisone 40 mg/day in addition to topical corticosteroids. Although transient improvement was observed, disease activity continued to increase, with the development of numerous bullae, including hemorrhagic lesions.

Symptoms persisted for several weeks. Because of her high-output stoma, history of malignancy, and increased risk of infection, immunosuppressive agents such as azathioprine, cyclosporin, and methotrexate were considered unsuitable. Off-label treatment with dupilumab was initiated in February 2024. However, blistering continued to progress, and BP180 IgG titers were elevated at 1:640 IU/mL.

Given the severe and refractory disease course, therapeutic plasma exchange (TPE) was initiated using the Spectra Optia^®^ Apheresis System (Terumo BCT, Lakewood, CO, USA). Treatment consisted of five sessions, performed daily for two consecutive days, followed by a 1-day interval and three additional daily sessions. Exchange volumes ranged from 1.0 to 1.5 plasma volumes per session. Clinical improvement became evident after two sessions, with re-epithelialization of lesions and a marked reduction in new blister formation (Fig. 1b, c).

To maintain remission, IVIg was administered at 0.45 g/kg/day for five consecutive days. A second cycle was given 4 weeks later at the same dosage. Within 2 weeks of initiating extracorporeal therapy, the patient reported complete resolution of symptoms (Fig. 1d). Disease control was subsequently maintained, allowing gradual tapering of prednisone to 10 mg/day.

### Relapse and GMA

Six months later, the patient experienced a severe relapse characterized by progressive, painful, disseminated blistering despite treatment with corticosteroids, IVIg, and ongoing dupilumab therapy, necessitating rehospitalization. BP180 IgG titers had increased to 1:2,560 IU/mL. Marked neutrophilia and elevated C-reactive protein levels suggested a pronounced inflammatory state. Concomitant bacterial superinfection was strongly suspected based on the clinical presentation and elevated inflammatory markers; however, no microbiological evidence was obtained to confirm the diagnosis (Fig. 1e).

Given the previous favorable response, a second course of TPE was initiated. However, no meaningful clinical improvement was observed after three sessions. In view of the lack of early response and concern that persistent inflammatory activity was contributing substantially to disease progression, TPE was discontinued. Considering the marked inflammatory phenotype and the recognized role of activated granulocytes and monocytes in BP pathogenesis, GMA was subsequently initiated using the Adacolumn^®^ device (JIMRO, Takasaki, Japan). Treatments were performed on alternate days for more than 180 min per session with heparin anticoagulation (5,000 IU at 6 mL/h).

After three GMA sessions, a marked reduction in blister formation and inflammatory skin activity was observed, enabling hospital discharge (Fig. 1f). No coagulation abnormalities occurred during treatment. Fibrinogen levels increased gradually throughout GMA, consistent with the absence of plasma protein removal.

### Outcome and Follow-Up

Disease activity was monitored clinically and serologically. During the first severe relapse in February 2024, BP180 IgG titers measured 1:640 IU/mL. At the time of the second relapse in September 2024, titers had increased fourfold to 1:2,560 IU/mL, coinciding with extensive blistering and elevated inflammatory markers. Following GMA, sustained clinical remission was accompanied by a decline in BP180 IgG levels to 1:160 IU/mL. Given the concurrent therapies and the fluctuating nature of BP, a causal relationship between GMA and the observed serological improvement cannot be established.

Although fluctuations in BP180 IgG titers paralleled disease activity in this patient, it should be noted that BP180 levels are not universally reliable markers of disease severity. Following the second relapse, dupilumab was continued as maintenance therapy. Corticosteroids were gradually tapered under close clinical monitoring, and the patient remained in sustained remission throughout follow-up.

## Discussion

This case illustrates the potential role of extracorporeal therapies in the management of severe, refractory BP, particularly in the setting of pronounced systemic inflammation and when conventional immunosuppressive strategies are limited by comorbidities. In our patient, a history of malignancy, high-output short bowel syndrome, and increased infection risk restricted the use of several established steroid-sparing agents, including azathioprine, cyclosporin, and methotrexate. Furthermore, impaired intestinal absorption may have limited the effectiveness of orally administered therapies.

TPE was associated with rapid clinical improvement during the first severe relapse and was followed by maintenance therapy with IVIg, resulting in sustained disease control for approximately 6 months. TPE enables the removal of circulating pathogenic autoantibodies, immune complexes, and inflammatory mediators, thereby reducing antibody-mediated tissue injury. In severe BP, extracorporeal antibody removal may be particularly attractive when rapid disease control is required or when prolonged exposure to high-dose corticosteroids is undesirable [9].

The role of extracorporeal therapies in BP has been increasingly recognized. Holtsche et al. [10] reported that immunoadsorption can be considered a relatively safe and effective adjunctive treatment in patients with severe or refractory BP. Similarly, recent updates on autoimmune bullous diseases have highlighted an expanding therapeutic armamentarium, including the recent FDA approval of the IL-4/IL-13 inhibitor dupilumab for moderate-to-severe BP, as well as the use of rituximab, IVIg, and immunoadsorption in selected refractory cases [11–14]. Nevertheless, refractory BP remains a major therapeutic challenge, underlining the ongoing need for effective and safe treatment alternatives [11].

In contrast to TPE, which non-selectively removes circulating plasma proteins including immunoglobulins and coagulation factors, GMA selectively targets activated myeloid cells without requiring plasma removal. The rationale for GMA in BP is supported by increasing evidence that innate immune mechanisms contribute substantially to disease pathogenesis. Although autoantibodies against BP180 and BP230 initiate the inflammatory cascade, effector cells and cell-mediated immune responses are also critical mediators of tissue injury [15].

More than 3 decades ago, Czech et al. hypothesized that activated granulocytes, through the release of eosinophil cationic protein, myeloperoxidase, and other granular contents, play an important role in blister formation and may serve as markers of disease activity [16]. More recently, a correlation between circulating neutrophil counts and disease severity has been reported, further supporting the importance of these cells in BP pathogenesis [15]. In addition, de Graauw et al. demonstrated that synergistic interactions between neutrophils and monocytes contribute to dermal-epidermal separation, suggesting that interruption of myeloid cell-mediated inflammatory pathways may represent a promising therapeutic strategy [17].

GMA specifically targets activated granulocytes and monocytes. The Adacolumn^®^ selectively adsorbs Mac-1-expressing neutrophils and monocytes through interactions involving complement fragment iC3b and CD11b expressed on activated leukocytes [18]. Consequently, GMA may modulate inflammatory pathways that contribute directly to blister formation and tissue damage. Although its therapeutic effects are generally attributed to depletion and functional modulation of activated myeloid cells, current evidence suggests that its clinical efficacy cannot be explained solely by leukocyte removal, and the precise immunomodulatory mechanisms remain incompletely understood [18].

In recent years, GMA has been increasingly explored in autoimmune and autoinflammatory dermatologic diseases. Gnesotto et al. [7] reviewed its use in conditions including pyoderma gangrenosum, palmoplantar pustular psoriasis, Sweet’s syndrome, adult-onset Still’s disease, impetigo herpetiformis, reactive arthritis, hidradenitis suppurativa, and cutaneous vasculitis, generally after failure of conventional therapies. GMA is also used in selected inflammatory conditions such as generalized pustular psoriasis, Behçet’s disease, and systemic lupus erythematosus [19] However, neither these reviews nor the current dermatologic literature report its use in BP [11].

To evaluate the novelty of our observation, a focused literature search of PubMed (last search: June 2026) was performed using the terms “bullous pemphigoid,” “granulocyte-monocyte apheresis,” “GMA,” “apheresis,” and “refractory bullous pemphigoid,” supplemented by screening the reference lists of relevant publications. No previously published reports describing the use of GMA in BP were identified. To the best of our knowledge, this appears to be the first published report of GMA use in refractory BP.

During the second relapse, characterized by severe blistering, neutrophilia, elevated inflammatory markers, and suspected superinfection, a second course of TPE failed to produce an early clinical response. After GMA initiation, significant clinical improvement was observed, permitting gradual corticosteroid tapering while maintaining remission, which remained sustained throughout follow-up.

Nevertheless, the differential response observed during the two relapses raises the possibility that extracorporeal therapies may exert distinct effects depending on the prevailing disease phase and inflammatory profile. While antibody-directed approaches such as TPE may be particularly effective during phases dominated by humoral autoimmunity, therapies targeting activated myeloid cells, such as GMA, may have additional value in inflammatory disease states characterized by neutrophil and monocyte activation [15, 17].

GMA may also offer theoretical safety advantages compared with plasma exchange as it does not require plasma removal and is therefore less likely to affect coagulation factors or serum protein levels. However, robust comparative safety data are lacking, and the optimal timing, patient selection, and sequencing of extracorporeal therapies in BP remain unknown. Given the single-patient nature of this observation and the concomitant administration of corticosteroids, IVIg, dupilumab, and plasma exchange, no conclusions regarding the efficacy of GMA can be drawn. Nevertheless, this case provides a rationale for further investigation of GMA in selected patients with refractory BP.

Overall, this case suggests that tailored extracorporeal approaches may represent a valuable therapeutic option in selected patients with severe, treatment-refractory BP. Prospective studies and larger observational cohorts are needed to clarify the respective roles of TPE and GMA and to determine whether specific clinical or immunological phenotypes may predict response to these therapies. The authors have completed the CARE reporting checklist, and the manuscript was prepared and revised in accordance with the CARE guidelines.

## Acknowledgment

We thank the patient for providing informed consent and for allowing her case to be reported for educational and scientific purposes.

## Statement of Ethics

This manuscript describes a single case report. Ethical approval was not required in accordance with applicable ethical and regulatory guidelines. Written informed consent was obtained from the patient for publication of the details of this medical case and any accompanying images.

## Conflict of Interest Statement

The authors have no conflicts of interest to declare.

## Funding Sources

Medical writing support was initially provided by Adacyte Therapeutics. The authors subsequently prepared and finalized the manuscript independently. Following acceptance of the manuscript, the article processing charge was covered by Adacyte Therapeutics. Adacyte Therapeutics had no role in the clinical management of the patient, data collection, data analysis or interpretation, preparation of the final manuscript, or the decision to submit the manuscript for publication.

## Author Contributions

Erich Reichinger: writing – original draft, writing – review and editing, conceptualization, data curation, methodology, resources, and software. Georgiana Ciobanu: writing – original draft, writing – review and editing, revision, conceptualization, data curation, methodology, resources, and software. Ieva Saulite: writing – review and editing, conceptualization, data curation, supervision, validation, and formal analysis. Antonio Cozzio: writing – review and editing, conceptualization, data curation, supervision, validation, and investigation. Norbert Arn: writing – review and editing, conceptualization, data curation, revision, supervision, validation, investigation, project administration, and formal analysis.

## Data Availability

The data that support the findings of this case report are not publicly available due to privacy reasons but are available from the corresponding author upon reasonable request.
